# Regulation of autophagy protects against liver injury in liver surgery‐induced ischaemia/reperfusion

**DOI:** 10.1111/jcmm.16943

**Published:** 2021-10-08

**Authors:** Chenxia Hu, Lingfei Zhao, Fen Zhang, Lanjuan Li

**Affiliations:** ^1^ Collaborative Innovation Center for the Diagnosis and Treatment of Infectious Diseases State Key Laboratory for the Diagnosis and Treatment of Infectious Diseases The First Affiliated Hospital Zhejiang University School of Medicine Hangzhou China; ^2^ National Clinical Research Center for Infectious Diseases The First Affiliated Hospital Zhejiang University School of Medicine Hangzhou China; ^3^ Key Laboratory of Kidney Disease Prevention and Control Technology Kidney Disease Center Institute of Nephrology The First Affiliated Hospital Zhejiang University School of Medicine Hangzhou China

**Keywords:** autophagy, cell death, ischaemia/reperfusion, liver resection, liver transplantation

## Abstract

Transient ischaemia and reperfusion in liver tissue induce hepatic ischaemia/reperfusion (I/R) tissue injury and a profound inflammatory response in vivo. Hepatic I/R can be classified into warm I/R and cold I/R and is characterized by three main types of cell death, apoptosis, necrosis and autophagy, in rodents or patients following I/R. Warm I/R is observed in patients or animal models undergoing liver resection, haemorrhagic shock, trauma, cardiac arrest or hepatic sinusoidal obstruction syndrome when vascular occlusion inhibits normal blood perfusion in liver tissue. Cold I/R is a condition that affects only patients who have undergone liver transplantation (LT) and is caused by donated liver graft preservation in a hypothermic environment prior to entering a warm reperfusion phase. Under stress conditions, autophagy plays a critical role in promoting cell survival and maintaining liver homeostasis by generating new adenosine triphosphate (ATP) and organelle components after the degradation of macromolecules and organelles in liver tissue. This role of autophagy may contribute to the protection of hepatic I/R‐induced liver injury; however, a considerable amount of evidence has shown that autophagy inhibition also protects against hepatic I/R injury by inhibiting autophagic cell death under specific circumstances. In this review, we comprehensively discuss current strategies and underlying mechanisms of autophagy regulation that alleviates I/R injury after liver resection and LT. Directed autophagy regulation can maintain liver homeostasis and improve liver function in individuals undergoing warm or cold I/R. In this way, autophagy regulation can contribute to improving the prognosis of patients undergoing liver resection or LT.

## 1 INTRODUCTION

Ischaemia/reperfusion (I/R) initiates a process with transient ischaemia and reperfusion that maintains liver tissue in a microenvironment characterized by anoxia and reoxygenation, which subsequently leads to tissue injury and a profound inflammatory response.^1^ Hepatic I/R injury in individuals or animals undergoing liver resection or liver transplantation (LT) may result in liver failure with high mortality. Both partial hepatectomy (PH) and LT are effective strategies for treating various liver diseases; however, LT is the only effective strategy for rescuing patients with end‐stage liver disease or irreversible liver malignant tumours.^2^ Notably, PH and orthotopic LT cause severe complications because they can initiate cell death and hepatic I/R injury. A large number of liver cells die, inducing hypohepatia or even liver failure. In addition, hepatic I/R plays a vital role in the pathophysiology of ischaemic‐type biliary lesions since a large number of cholangiocytes undergo apoptosis and necrosis.^3^

**FIGURE 1:**
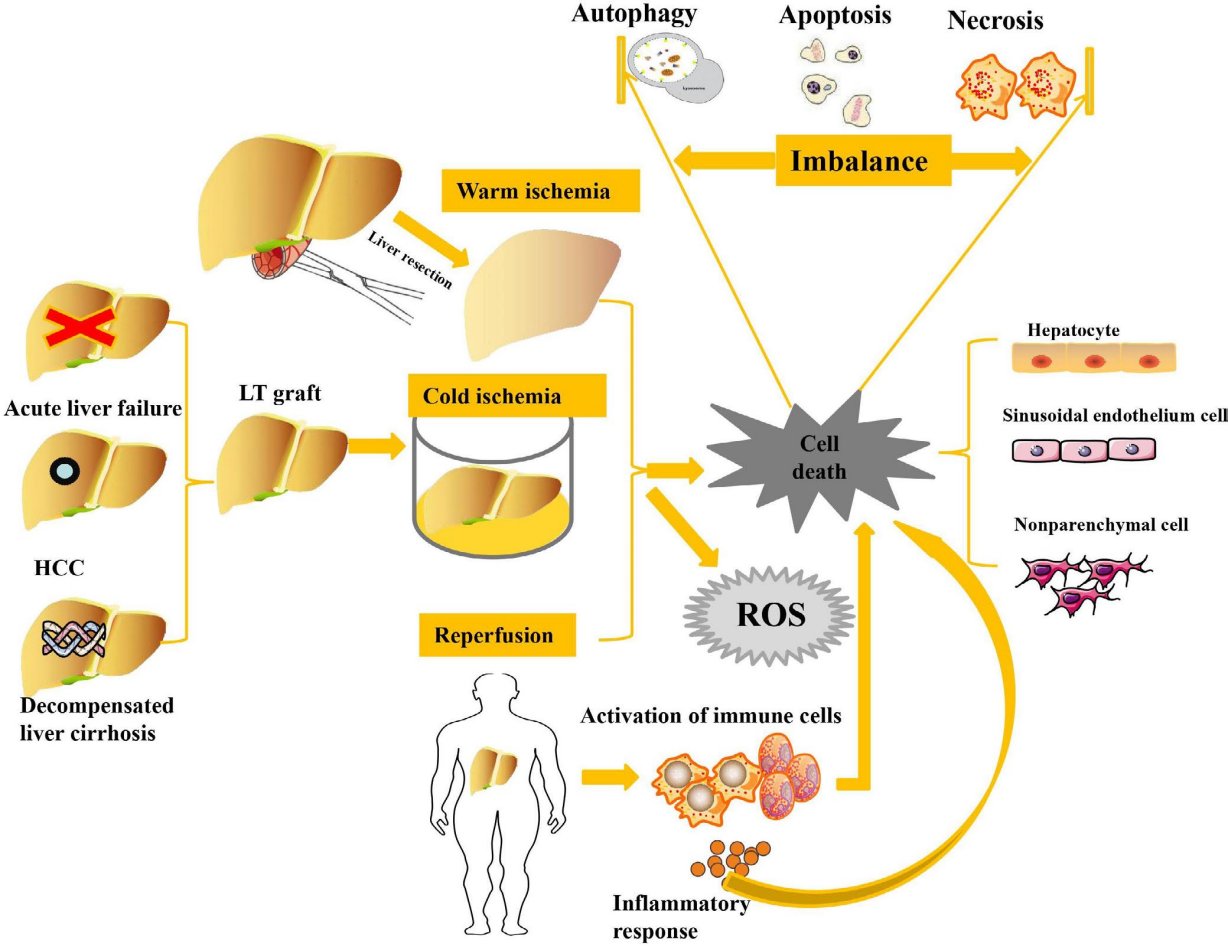
Warm I/R or cold I/R initiates three types of cell death: apoptosis, necrosis and autophagy. Hepatic I/R also induces ROS accumulation, the immune response and inflammation at injury sites, which in turn aggravates the death of hepatocytes, sinusoidal endothelial cells and nonparenchymal cells

In general, I/R can be classified into two types: warm I/R or cold I/R (Figure 1). Warm I/R is observed in patients or animal models undergoing liver resection, haemorrhagic shock, trauma, cardiac arrest or hepatic sinusoidal obstruction syndrome when vascular occlusion inhibits normal blood perfusion in liver tissue.^4^ Cold I/R is a condition affecting only patients who have undergone LT because the donated liver graft is preserved in a hypothermic environment prior to entering a warm reperfusion phase.^5^ Warm ischaemia induces nutrient depletion, adenosine triphosphate (ATP) depletion and anaerobic glycolysis and promotes the generation of an acidic milieu in the cytoplasm, which subsequently suppresses a myriad of protective enzymes. Although a harsh acidic environment can protect against injury in the liver parenchyma during an acute ischaemic event, severe liver acidosis eventually results in the death of primary hepatocytes after the activation of apoptosis‐ or necrosis‐related pathways.^6^ On the other hand, cold ischaemia mainly induces cell death in the sinusoidal endothelium and nonparenchymal cells in liver tissue.^7^, ^8^ Although hypothermia has been shown to reduce energy metabolism and preserve the function of liver grafts, a prolonged ischaemia period and hypothermia lead to cell swelling and liver injury after damaging Na/K ATPase membrane pumps, leading to the accumulation of sodium and chloride.^9^, ^10^ Hypothermia has also been shown to induce a strong adaptive immune response in resected liver grafts characterized by recruitment of T cells into the ischaemic graft.^11^ Similarly, liver reperfusion‐induced inflammation initiates an innate immune‐dominant response in conventional T lymphocytes, which subsequently leads to injury of both parenchymal and nonparenchymal cells in situ and in liver transplants.^12^, ^13^ Moreover, other inflammatory cells are recruited in the response to hepatic I/R after restoration of blood flow and pH neutralization.^8^ In the early stage of reperfusion, resident hepatic macrophages are activated to induce reactive oxygen species (ROS) generation and oxidative stress, while neutrophils are recruited to release inflammatory factors and induce tissue damage at the late stage of reperfusion.^14^ Liver I/R also upregulates the release of a cascade of inflammatory factors, including tumour necrosis factor‐α (TNF‐α), interleukin (IL)‐1, IL‐2, IL‐6 and high mobility group box 1 (HMGB1).^7^ Mitochondrial ROS, immune responses and inflammatory factor activation lead to further activation of cell death‐related pathways.

Apoptosis, necrosis and autophagy are three main types of cell death in rodents and patients with warm or cold I/R injury. The initiation of apoptosis, necrosis and autophagy may be induced by similar effectors and is usually regulated by similar signalling pathways.^15^ Hepatic I/R activates phagocytosis and degradation of apoptotic bodies after cells undergo cell shrinkage, nucleus condensation, chromatin margination and fragmentation of both the nucleus and cytoplasmic structures. This process is determined as apoptosis in cells with normal in appearance. Physiologically, apoptosis is critical for the elimination of damaged or senescent cells and tissue remodelling in injury environments.^16^ Apoptosis is initiated either by extrinsic stimuli through cell surface death receptors, including TNF‐α, Fas and TNF‐related apoptosis‐inducing ligand (TRAIL) receptors, or by intrinsic stimuli via a mitochondrial pathway.^17^ Thus, activation of caspases leads to cell structure destruction and apoptosis.^18^ Compared to apoptosis, pathological necrosis is a disordered and passive cell death in response to acute injury that involves cell bursting but is not generally triggered in normal development. Necrosis progression is typically not associated with the activation of caspases, and the cellular nucleus becomes distended and remains largely intact.^16^, ^19^ The morphologic feature of necrosis is elimination of organelles, formation of large plasma membrane blebs, bleb rupture and subsequent loss of the plasma membrane permeability barrier.^20^ Rupture of the plasma membrane promotes the release of cellular contents into the extracellular environment and induces a significant inflammatory response.^21^ Under physiological conditions, a relatively low level of autophagy plays a homeostatic role in maintaining cell structures and functions by recycling long‐lived proteins and whole organelles.^22^ Under stress conditions, autophagy plays a critical role in promoting cell survival and maintaining liver homeostasis by generating new ATP and organelles after degradation of macromolecules and organelles in liver tissue. Activation of autophagy inhibits the progression of caspase‐dependent apoptosis, and the activation of caspase‐dependent apoptosis inhibits the autophagic process. However, under some conditions, autophagy proceeds to autophagic cell death, which promotes the progression of apoptosis or necrosis in mammals.^23^, ^24^

Recent studies have highlighted therapies targeting the suppression of I/R injury in liver tissue that rely on complex mechanisms; however, there is a particular emphasis on therapies that control autophagy in liver tissue to reduce inflammation and organ failure. In this review, we comprehensively discuss current strategies and the underlying mechanisms for alleviating I/R injury in liver resection and LT via autophagy regulation. We hope that more investigators will seek to control autophagy regulation specifically to maintain liver homeostasis and improve liver function in individuals undergoing warm or cold I/R. In this way, autophagy regulation will contribute to improving the prognosis of patients undergoing liver resection or LT.

## 2 AUTOPHAGY IN LIVER TISSUE

Three main autophagy‐related pathways participate in the degradation of cytosolic contents in lysosomes. Macroautophagy facilitates the degradation of entire organelles or parts thereof after the development of a double‐membrane autophagosome and the subsequent generation of autolysosomes that degrade cytoplasmic material.^25^ Macroautophagy is selectively initiated by organelle injury, and it is upregulated nonspecifically through bioenergetic instability.^26^ Microautophagy, which is mediated by acidic organelles such as late endosomes, is the least studied type of autophagy.^27^ It is initiated by amino acid starvation.^28^ Microautophagy decomposes small autophagic substrates by packaging a cytoplasmic portion into the lumen of a lysosome after the lysosomal membrane is rearranged and develops a protruding arm‐like structure.^29^ Chaperone‐mediated autophagy (CMA) degrades only soluble proteins with a KFERQ motif after binding to heat shock protein family A member 8 (HSPA8) and does not involve vesicle invagination, while biodegradable substrates enter the lysosomal lumen facilitated by a specific spliced isoform of lysosomal‐associated membrane protein 2 (LAMP2A).^25^ Several injury‐causing factors, such as DNA damage, hypoxia and oxidative stress, significantly induce the activation of CMA in mammals.^30^, ^31^, ^32^ Moreover, CMA is important in the regulation of liver homeostasis because it maintains hepatic metabolism and inhibits tumorigenesis.^25^

Macroautophagy is the predominant form of autophagy and is often referred to as autophagy. Wang et al. highlighted findings showing that reduced autophagic flux is able to impair the adaptive capacity of cells or tissues, enabling them to withstand oxygen damage, including that caused by ischaemia or hypoxia.^33^ Komatsu et al. demonstrated that autophagy‐related protein (ATG)7 deficiency resulted in enlarged livers in up to 30% of the body of mice and abnormal structures in mitochondria and peroxisomes of hepatocytes.^34^ Disabled autophagy in hepatocytes may induce severe hepatic injury since the limited half‐life of primary hepatocytes leads to the accumulation of detrimental cellular byproducts.^35^ Moreover, autophagy upregulation is initiated to inhibit ROS‐induced hepatocellular necrosis after the expression levels of microtubule‐associated protein 1 light chain (LC)3‐II is upregulated, increasing the number of autophagosomes in liver tissue under partial warm ischaemia without reperfusion. However, inhibition of autophagy by chloroquine treatment aggravates mitochondrial oxidative stress and mitochondrial dysfunction.^36^ Zhu et al showed that rapamycin (an autophagy activator) effectively downregulated endoplasmic reticulum (ER) stress, inhibited the mechanistic target of rapamycin (mTOR) pathway and enhanced autophagy to protect against liver injury induced by I/R.^37^ However, others have debated whether autophagy activation initiates or aggravates hepatic I/R injury by enhancing liver inflammation. Rapamycin was reported to inhibit liver regeneration and liver weight reconstitution, accompanied by upregulation of inflammatory factors, including TNF‐α and IL‐1Ra, but downregulation of hepatocyte growth factor (HGF) and angiogenesis‐related factors, including vascular endothelial growth factor receptor 2 (VEGFR2) and angiopoietin, in mice after PH.^38^

**FIGURE 2:**
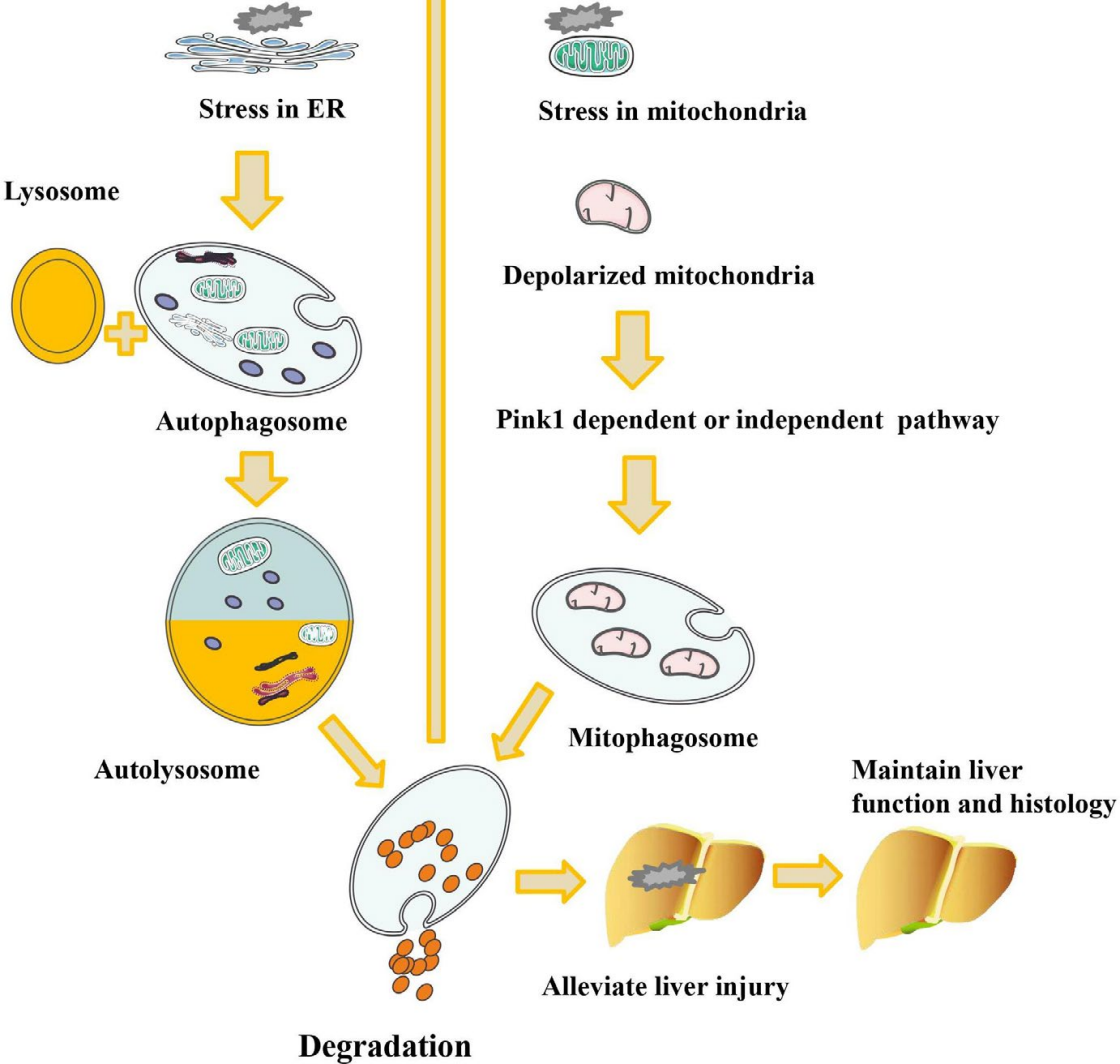
Autophagy and mitophagy contribute to the degradation of organelles to maintain liver function and histology and repair liver injury in hepatic I/R

Currently, expert opinion suggests that macroautophagy can be further classified into nonselective autophagy and selective macroautophagy, which targets special organelles or specific compounds for degradation. It is widely accepted that macroautophagy is able to degrade various organelles or substrates, such as mitochondria, the ER, peroxisomes, ribosomes, lipid droplets, iron‐based compounds, glycogen, protein aggregates and cytoplasmic pathogens.^25^ Specific names have been ascribed to autophagy of specific compounds, including mitophagy (mitochondria), reticulophagy (ER), pexophagy (peroxisomes), ribophagy (ribosomes), lipophagy (lipids) and ferritinophagy (iron‐based compounds).^39^ Reticulophagy effectively maintains the structure and function of the ER via the interaction of diverse receptors with LC3 to generate autophagosomes under various conditions, including starvation, nonalcoholic fatty liver disease (NAFLD), viral infection and fibrosis.^39^ Lipophagy is another important macroautophagic form that is involved in lipid homeostasis and metabolism in liver diseases such as alcoholic liver and nonalcoholic liver diseases, including fibrosis, cirrhosis and hepatocellular carcinoma.^39^ Notably, mitophagy plays a critical role in removing worn‐out mitochondria, which have a half‐life of 10 to 25 d in healthy liver.^40^ Mitophagy is another important mechanism in which selective abnormally cleaved proteins or damaged mitochondria are eliminated to inhibit the accumulation of mitochondrial‐derived ROS and mutated mitochondrial DNA (Figure 2).^41^ Hepatic I/R triggers mitophagy via two distinct pathways: phosphatidylinositol‐3‐kinase (PI3K)‐dependent and PI3K‐independent signalling pathways.^42^ Kim et al^43^ demonstrated that inhibition of mitophagy resulted in accumulation of dysfunctional mitochondria, uncoupling of oxidative phosphorylation, ATP depletion and cell death in liver I/R models. Abrogated or inefficient mitophagy may result in uncontrolled ROS accumulation, energy metabolism failure and ultimately cell death in liver tissue.

## 3 AUTOPHAGY REGULATION ATTENUATES WARM HEPATIC I/R INJURY

Many previous studies have shown that autophagy upregulation or downregulation exerts protective effects in the attenuation of warm hepatic I/R injury according to the specific circumstances (Table 1). Starvation or ischaemic preconditioning, chemical exposure, plant extract treatment, MSC and/or MSC derivative application, and gene modification contribute to maintaining liver homeostasis and liver function in warm hepatic I/R models and patients via autophagy regulation and related mechanisms.

**TABLE 1:** Autophagy upregulation or downregulation exerts protective effects in attenuating warm hepatic I/R injury

Animal	Time	Treatment	Autophagy regulation	Effect	Mechanism	Ref.
Mouse	Pretreatment	Short‐term starvation	↑	Decrease serum aminotransferases levels; decrease hepatocyte apoptosis; attenuate pathological damage	Upregulate the expression of SIRT1; inhibit hepatocellular apoptosis	44
Mouse	Pretreatment	IP	↑	Alleviate liver I/R injury; decrease serum levels of aminotransferases, inflammatory cytokines; reduce histopathologic changes	Activate autophagy and the HO‐1‐mediated signalling pathway	45
Mouse	Pretreatment	RIP	↑	Decrease the incidence of hepatocyte necrosis, ballooning degeneration and sinusoidal congestion; reduces Suzuki's score	Increase HO‐1 expression; activate the ERK1/2 and p38‐MAPK pathways	46
Mouse	Pretreatment	Cisplatin	↑	Reduce I/R‐induced ALT level and liver damage	Prevent the release of HMGB1; decrease liver I/R‐induced inflammatory mediator production	49
Mouse	Pretreatment	ATRA	↑	Diminish levels of ALT and AST; decrease the degree of histopathological changes	Inhibit apoptosis and inflammation	50
Mouse	Pretreatment	Amiodarone	↑	Improve hepatocyte proliferation; increase the survival rate of PH mice	Remove damaged mitochondria; mTOR‐independent signalling	51
Mouse	Pretreatment	ATG7 knockdown or chloroquine	↓	Aggravated liver injury; reduce liver growth and hepatocyte proliferation	Increase the number of dysfunctional mitochondria	51
Rat	Pretreatment	Lithium	↑	Decrease levels of ALT and AST	Decrease neutrophil Infiltration; decrease production of inflammatory mediators; decrease HMGB1 expression and release; prevent dephosphorylation of GSK‐3β; decrease hepatic apoptosis	52
Mouse	Pretreatment	Spermidine	↑	Decrease levels of serum transaminases; decrease release of inflammatory cytokines; inhibit histopathological changes	Inhibit liver apoptosis; alleviate I/R‐induced inflammation; activate the AMPK‐mTOR‐ULK1 signalling pathway	53
Mouse	Pretreatment	NAC	↑	Decrease levels of ALT and AST; attenuate pathological changes	Attenuate JNK‐mediated signalling pathway activation; inhibit the expression of Bax, TNF‐α, NF‐κB, IL2, IL6;	54
Mouse	Pretreatment	Vitamin D	↑	Preserve liver function; reduce histological damage	Ameliorate oxidative stress; attenuate inflammation; decrease the levels of TNF‐α, IL‐6 and IL‐2; increase the IL‐10 level	55
Mouse	Pretreatment	Antecedent tri‐iodothyronine	↑	Preserve liver function; decrease apoptosis rate; reduce histological damage	Upregulate the MEK/ERK/mTORC1 signalling pathway; enhance antioxidative stress; attenuate neutrophil infiltration	56
Rat	Pretreatment	Baicalein	↑	Blunt elevation of ALT and AST levels; attenuate histological changes	Upregulate HO‐1 expression	57
Mouse	Pretreatment	Shikonin	↓	Reduce serum AST and ALT levels; improve pathological features; reduce hepatocyte apoptosis rate	Decrease the expression of Bax, caspase 3 and caspase 9; reduce the release of IL‐1β, TNF‐α and IL‐6; activate the PI3K/Akt signalling pathway	58
Mouse	Pretreatment	OA	↑	Improve tolerance to hepatic I/R injury	Decrease phosphorylation of AKT; decrease p65 nuclear translocation; downregulate oxidative stress	59
Mouse	Pretreatment	OA	↓	Downregulate ALT and AST levels; improve histology	Inhibit phosphorylation of JNK; decrease the expression of HMGB1 and TNF‐α; inhibit apoptosis	60
Mouse	Pretreatment	Bergenin	↓	Alleviate liver function injury; inhibit liver IR‐induced apoptosis	Eliminate ROS; inhibit the release of inflammatory factors; activate the PPAR‐γ signalling pathway	61
Mouse	Pretreatment	Salidroside	↓	Ameliorate hepatic I/R injury	Downregulate phosphorylation of P38, JNK and ERK to suppress pathway activation; inhibit the MAPK signalling pathway; inhibit the release of IL‐6 and TNF‐α; reduce the hepatocyte apoptosis rate	62
Mouse	Pretreatment	Astaxanthin	↓	Ameliorate hepatic I/R injury involving liver enzyme pathology	Inhibit the release of ROS and inflammatory cytokines; reduce the release of inflammatory factors including TNF‐α and IL‐6; reduce the Bax/Bcl‐2 ratio; attenuate ROS/MAPK signalling pathway activation	63
Mouse	Pretreatment	Quercetin	↓	Ameliorate hepatic I/R injury; inhibit apoptosis	Inactivate the ERK/NF‐*κ*B signalling pathway; inhibit the release of TNF‐α and IL‐6	64
Mouse	Pretreatment	Levo‐tetrahydropalmatine	↓	Alleviate I/R injury	Inhibit ERK/NF‐κB‐mediated hepatocyte apoptosis; downregulate the release of TNF‐α and IL‐6	65
Rat	Pretreatment	MSCs	↑	Decrease hepatocellular necrosis and cytoplasmic vacuolization; decrease ALT and AST levels	Upregulate HO‐1 expression	67
Porcine	Posttreatment	MSCs	↓	Decrease serum levels of AST, ALT, total bilirubin and lactate dehydrogenase	Attenuate oxidative stress; suppress the generation of myeloperoxidase and malondialdehyde	68
Mouse	Posttreatment	Rapamycin pretreated MSCs	↑	Enhance recovery of hepatic function; attenuate pathological changes	Attenuate the inflammatory response; reduce oxidative stress; enhance MSC homing via the CXCR4/CXCL12 axis	69
Rat	Posttreatment	HSP‐MSCs	↑	Improve histopathology; reduce Suzuki scores	Increase the homing and survival rate of transplanted MSCs	70
Mouse	Pretreatment and posttreatment	MSC‐Heps‐Exo	↑	Decrease serum levels of liver enzymes	Reduce hepatocyte apoptosis rate	71
Mouse	Pretreatment	KO of ATG5	↓	Impair recovery of liver regeneration; attenuate DNA synthesis; decrease liver hypertrophy and hepatocyte senescence	Suppress autophagy activity and mitochondrial oxidation	72
Rat	Pretreatment	KO of parkin	↓	Aggravate DNA damage; promote hepatocyte apoptosis, reduce hepatocyte survival rate	Suppress Bcl‐2; induce cell cycle arrest	73
Mouse	Pretreatment	Overexpression of miR‐1907	↑	Increase the liver/body weight ratio	Promote liver regeneration; target the GSK3β signalling pathway	74
Mouse	Pretreatment	Overexpression of miR‐17	↑	Aggravate hepatic I/R injury	Diminish Stat3 and p‐Stat3 levels	75
Mouse	Pretreatment	KO of ASPP2	↓	Enhance liver regeneration	Upregulate the mTORC1 signalling pathway	76

### 3.1 Starvation or ischaemic preconditioning

I/R initiates liver injury through transient ischaemia and reperfusion; intriguingly, preconditioning tissue with starvation or ischaemia effectively protects against liver injury. Short‐term starvation through calorie restriction or fasting has been shown to exert beneficial effects on multiple organs and preserve organ function under stress conditions. Short‐term starvation protects against liver I/R injury, as evidenced by lower levels of serum aminotransferases and hepatocyte apoptosis and improved pathological damage in mouse models. Furthermore, starvation significantly upregulated hepatocellular autophagy and the expression of the longevity gene silent information regulator factor 2‐related enzyme 1 (SIRT1) in I/R‐treated liver tissue.^44^ Investigators pretreated mice with transient ischaemia (10–15 min) in liver tissue around the portal triad by occluding of hepatic inflow by applying a vascular clamp, and then, they removed the clamp to cause transient reperfusion (10–15 min). Ischaemic preconditioning (IP) significantly activated autophagy and the haem oxygenase (HO)‐1‐mediated pathway to ameliorate hepatic I/R injury, as indicated by the downregulation of aminotransferases and inflammatory cytokine release and the reduction in histopathologic changes in the liver tissue.^45^ Remote ischaemic preconditioning (RIPC) is defined as transient ischaemia in distant tissues or organs such as a limb or intestine to protect liver tissue from I/R‐induced injury. RIPC is applied to limbs through induced ischaemia for dozens of minutes, followed by dozens of minutes of reperfusion prior to long‐term ischaemia. RIPC has been shown to protect the liver from I/R injury by inducing HO‐1/p38‐mitogen‐activated protein kinase (MAPK)‐dependent autophagy.^46^ Although various experimental studies showed pronounced benefits of IP for liver surgery, a meta‐analysis showed that IP failed to improve the prognosis of patients after liver resection or LT.^47^, ^48^

### 3.2 Chemicals

Autophagy activation, inhibition of inflammation or inhibition of oxidative stress has been reported to improve liver function and the prognosis of I/R injury. During hepatic I/R, injured sites showed significantly upregulated the expression of Beclin‐1 and ATG8/LC3 indicating the formation and expansion of autophagosomes in vivo to protect against hepatic I/R‐induced injury. Administration of nontoxic concentrations of cisplatin notably increased mitophagy levels and prevented the release of HMGB1 induced by hepatic I/R, which subsequently attenuated liver injury.^49^ All‐trans retinoic acid (ATRA) is effective in protecting the liver from I/R injury by enhancing autophagy in a forkhead box class O (FOXO)3/p‐AKT/FOXO1 signalling‐dependent manner.^50^ Lin et al. demonstrated that amiodarone significantly improved hepatocyte proliferation, liver tissue growth and the survival rate of PH mice by upregulating autophagy and eliminating damaged mitochondria.^51^ Before 60 min of partial warm ischaemia, chronic lithium treatment significantly reduced I/R‐induced liver injury by inducing liver autophagy and reducing hepatic inflammatory cytokine levels, neutrophil infiltration and HMGB1 release.^52^ Similarly, spermidine pretreatment significantly attenuated liver apoptosis, the levels of serum transaminases and histopathological changes via upregulation of AMP‐activated protein kinase (AMPK)‐mTOR‐unc‐51 like kinase 1 (ULK1)‐dependent autophagy and downregulation of inflammatory cytokines.^53^ N‐acetylcysteine (NAC) has been reported to prevent I/R injury by inducing autophagy after attenuation of the c‐Jun N‐terminal kinase (JNK)‐mediated signalling pathway and downregulation of Bax, TNF‐α, nuclear factor‐kappaB(NF‐κB), IL‐2 and IL‐6.^54^ Vitamin D significantly preserved liver function and decreased histological damage via amelioration of oxidative stress and upregulation of autophagic flux. However, inhibition of the mitogen‐activated protein kinase kinase (MEK)/extracellular signal‐regulated kinase (ERK) pathway or phosphatase and tensin homolog (PTEN)/PI3K/AKT/mTOR pathway partially abolished the protective effect of vitamin D in hepatic I/R mice, and knockdown of Beclin‐1 completely reversed the protection conferred by vitamin D.^55^ On the other hand, antecedent tri‐iodothyronine injection significantly preserved liver function, decreased the apoptosis rate, reduced histological damage and enhanced antioxidative stress in hepatic I/R mice by upregulating MEK/ERK/mTORC1‐mediated autophagy.^56^ This study indicates that targeting autophagy regulation by chemicals is vital in the protection of hepatic I/R‐induced injury.

### 3.3 Plant extracts

A large amount of evidence has shown that activation of autophagy by plant extract treatment contributes to liver protection via the activation of different signalling pathways. Baicalein pretreatment blunted an increase in alanine transaminase (ALT) and aspartate aminotransferase (AST) and alleviated histological changes via induction of autophagy and upregulation of HO‐1 expression.^57^ Shikonin pretreatment reduced serum transaminase levels and attenuated pathological features by upregulating PI3K/AKT‐mediated autophagy but decreasing the expression of apoptosis‐related proteins, including Bax, caspase‐3 and caspase‐9.^58^ Pretreatment with oleic acid (OA) improved the tolerance of mice undergoing hepatic I/R injury by downregulating the phosphorylation of AKT and oxidative stress. An in vitro study showed that OA effectively inhibited oxidative stress, inflammation and cell death in hydrogen peroxide (H_2_O_2_)‐treated HepG2 cells by upregulating autophagy.^59^

Other investigations showed that plant extract pretreatment attenuated hepatic I/R injury via inhibition of autophagy and apoptosis. Pretreatment with OA for 7 consecutive days prior to PH notably alleviated liver injury by downregulating caspase‐3, caspase‐9, Bax, Beclin‐1 and LC3 and inhibiting JNK, HMGB1 and TNF‐α in hepatic I/R mice.^60^ Bergenin pretreatment significantly decreased the levels of liver enzymes by inhibiting autophagy and apoptosis, and reducing the levels of mitochondrial ROS and inflammatory factors such as TNF‐α, IL‐6 and IL‐1β. At the molecular level, bergenin pretreatment upregulated the PPAR‐γ pathway but downregulated the phosphorylation of P38 MAPK, NF‐κB p65 and Janus kinase 2 (JAK2)/signal transducer and activator of transcription 1 (STAT1) pathways in a dose‐dependent manner.^61^ Pretreatment with salidroside or astaxanthin significantly inhibited the release of ROS and inflammatory cytokines via inactivation of P38 MAPK, JNK and ERK and inhibition of autophagy and apoptosis in hepatic I/R mouse models.^62^, ^63^ Quercetin and levo‐tetrahydropalmatine (L‐THP) preconditioning also conferred protection against hepatic I/R injury and inhibited the release of inflammatory cytokines via inactivation of the ERK/NF‐*κ*B pathway and inhibition of autophagy and apoptosis.^64^, ^65^

### 3.4 MSCs and derivatives

Recently, the application of mesenchymal stem cells (MSCs) has been a hot topic in regenerative medicine used to treat of various diseases. These cells and their derivatives are able to enhance liver regeneration and liver function via their migration, differentiation, anti‐inflammation, antioxidation and immunoregulation capacities.^66^ MSCs mitigate I/R injury via upregulation of HO‐1 expression and autophagy in vivo, while the autophagy inhibitor 3‐methyladenine or HO‐1 zinc protoporphyrin IX significantly reverses the protective effects of MSCs.^67^ Ge et al^68^ documented that MSCs effectively enhanced liver function in PH porcine models by attenuating oxidative stress and autophagy, as demonstrated by the reduction in autophagy markers such as Beclin‐1, ATG5, ATG12 and LC3II and upregulation of P62. Rapamycin is able to enhance MSC migrative and anti‐inflammatory capacities without affecting MSC viability in vitro. Rapamycin pretreatment enhanced MSC migration into injured liver sites and subsequently improved hepatic performance, attenuated pathological changes and suppressed the release of inflammatory cytokines in a liver I/R mouse model.^69^ Heat shock pretreatment (HSP) for 2 h reduced the apoptosis rate of MSCs, as evidenced by downregulation of Bax and cytochrome C and upregulation of Bcl‐2 and autophagy in H_2_O_2_‐treated MSCs. Administration of HSP‐pretreated MSCs significantly decreased the levels of serum aminotransferases, lowered Suzuki scores and increased the survival rate in I/R animal models via activation of the p38MAPK/mTOR pathway.^70^ In addition to MSCs, their derivatives, such as hepatocyte‐like cells or exosomes, contribute to liver protection via autophagy regulation. MSC‐derived hepatocyte‐like cell exosomes (MSC‐Heps‐Exos) significantly inhibited hepatocyte apoptosis and reversed hepatic I/R injury in vitro and in vivo by upregulating autophagic flux.^71^

### 3.5 Gene modification

Multiple studies have demonstrated that gene modification with autophagy inhibition aggravates liver injury, while autophagy enhancement protects against I/R injury. Knockdown of ATG7 aggravates hepatic I/R injury and inhibits liver regeneration by abrogating autophagy and damaging mitochondria.^51^ ATG5 knockout (KO) promoted the release of senescence‐associated factors, including IL‐6 and IL‐8, and aggravated PH‐induced injury in mice by impairing autophagy and upregulating the expression levels of hepatic P62 and P21.^72^ Parkin KO induced cell cycle arrest and aggravated DNA damage via suppression of mitophagy and promotion of hepatocyte apoptosis, leading to reduced hepatocyte survival in a hepatic I/R model.^73^ Overexpression of miR‐1907 in mice led to a significantly higher liver weight/body weight ratio in PH‐treated mice by activating autophagy and enhancing hepatocyte proliferation and liver regeneration.^74^

However, several studies have demonstrated that activation of autophagy aggravates hepatic I/R injury, while inhibition of autophagy protects against PH‐induced injury. Overexpression of miR‐17 enhanced autophagic flux and activated the transcription‐3 pathway, subsequently aggravating hepatic I/R injury in animal models.^75^ Apoptosis‐stimulating protein two of p53 (ASPP2) KO significantly enhanced liver regeneration in mice with 70% PH by upregulating the mTORC1 pathway and downregulating the downstream autophagic pathway.^76^

## 4 AUTOPHAGY REGULATION AND LT PROGNOSIS

Liver grafts in animal models and patients undergoing LT are challenged by exposure to cold ischaemia/warm reperfusion (CI/WR) induced by changes in the organ environment. Storage of excised liver grafts in cold preservation solutions initiates cold ischaemia and liver injury, and warm reperfusion promotes the generation of abundant autophagosomes and autolysosomes, which subsequently dissociates and obstructs the sinusoid. The critical role of autophagy regulation in LT is the protection of liver grafts from I/R injury and rejection (Table 2). Then, upregulated autophagy results in massive necrosis of hepatocytes within 2 h without activation of caspase‐3 or caspase‐7. However, the PI3K inhibitors wortmannin and LY294002 effectively reduced liver damage and the mortality rate of recipient rats via suppression of autophagy.^77^ It has also been reported that autophagy inhibition contributes to the regulated activity of T cells and immune regulation in LT models. Chen et al. demonstrated that the autophagy inhibitor 3‐methyladenine (3‐MA) significantly prolonged the liver graft survival time of rats after LT by inhibiting the activation of CD8+ T cells. They indicated that inhibition of autophagy may be an effective strategy to improve the prognosis of patients after LT by decreasing rejection and improving immune tolerance.^78^

**TABLE 2:** Autophagy regulation protects liver grafts from I/R injury and rejection

Animal	Time	Treatment	Autophagy regulation	Effect	Mechanism	Ref.
Rat	Posttreatment	PI3K inhibitors, wortmannin or LY294002	↓	Reduce liver damage and the mortality rate of recipient rats	Inhibit autophagy	77
Rat	Pretreatment	3‐MA	↓	Prolong liver graft survival in rats after LT; reduce rejection	Reduce function of CD8+ T cells; accelerate the apoptosis of CD8+ T lymphocytes	78
Mice and human	Pretreatment	Antibiotic	↑	Improve hepatocellular function; decrease incidence of early allograft dysfunction	Downregulate CHOP and inflammation	79
Rat	Pretreatment	Berberine	↑	Restore liver function; preserve liver histology	Attenuate oxidative stress and apoptosis; activate the SIRT1/FoxO3a signalling pathway	80
Rat	Preatreatment	HO‐1‐overexpressing MSCs	↑	Decrease histopathological characteristics and rejection activity index of transplanted liver	Activate the ERK/mTOR signalling pathway	81
Mouse and human	Pretreatment	HO‐1‐overexpressing macrophages	↑	Decrease LT‐induced liver damage	Upregulate SIRT1/LC3B expression; promote anti‐inflammation; attenuate hepatocellular death; enhance SIRT1/LC3B expression	82

Antibiotics may serve as therapeutic targets in LT by upregulating autophagy and downregulating ER stress and inflammation. Antibiotic pretreatment attenuated hepatic I/R injury in mouse allogeneic OLTs via the gut‐liver axis. Nakamura et al. showed that antibiotic pretreatment improved liver function, decreased the incidence of early allograft dysfunction, and mitigated I/R injury in mice after LT by upregulating the levels of PGE2 receptor 4 (EP4) and LC3B but decreasing CCAAT/enhancer‐binding protein homologous protein (CHOP) levels.^79^ Berberine, a traditional Chinese medicine, restored liver function and preserved liver histology in rats after LT through the upregulation of SIRT1/FOXO3α‐mediated autophagy and attenuation of liver‐specific oxidative stress and apoptosis.^80^ HO‐1‐overexpressing MSCs exerted a particularly significant protective effect on liver grafts following reduced‐size liver transplantation (RSLT) via activation of the ERK signalling pathway and upregulation of autophagy.^81^ In addition to MSCs, other cell types that participate in regulating autophagy in vivo have been transplanted in mice after LT to enhance liver function. Nakamura et al. demonstrated that injection of HO‐1‐overexpressing macrophages into mice prior to LT significantly decreased LT‐induced liver damage by upregulating SIRT1/LC3B expression.^82^

## 5 AUTOPHAGY REGULATION IN LIVER DONATION‐AFTER‐CARDIAC DEATH (DCD) AND AGED OR STEATOTIC LIVER GRAFTS USED FOR LT

Insufficient liver graft donation is a major disadvantage in LT in the clinic; therefore, liver grafts donated after death or donated by elderly patients or steatotic liver patients can partially remedy the shortage in the clinic. However, these grafts are more sensitive to warm ischaemia, cold storage and reperfusion, which remarkably increase graft dysfunction and liver failure.^83^

### 5.1 Autophagy regulation in DCD liver with hepatic I/R injury

During hepatic I/R, animals showed higher levels of ALT and AST and accelerated autophagy, accompanied by upregulated glycogen synthase kinase‐3β (GSK‐3β) and AMPK expression in transplanted DCD liver tissue.^84^ However, Zeng et al^85^ argued that autophagy activation enhances the liver function of DCD liver grafts. In a DCD model, hypothermic oxygenated machine perfusion (HOPE) was used to perfuse 100% oxygen into liver tissue for 1 h. This perfusion remarkably decreased the hepatic levels of ALT, AST and ROS in donor grafts; moreover, it decreased the apoptosis and necrosis rates of hepatocytes by replenishing ATP and upregulating autophagy‐related proteins such as ULK1, ATG5 and LC3B‐II.

### 5.2 Autophagy regulation in aged liver grafts with hepatic I/R injury

Cell and tissue ageing is a normal phenomenon in elderly people, and it is closely related to the high incidence and severity of diseases in this population. Ageing has been associated with elevated levels of ALT, AST, lipofuscin accumulation, steatosis, fibrosis and defective liver regeneration after PH. In addition, ageing is often associated with reduced autophagic flux and lower liver regeneration capacity. In elderly patients, ageing liver tissue shows slow hepatic blood flow and decreased quantities of mitochondria and endoplasmic reticula, which results in poorer regeneration after PH and LT.^86^ In comparison to 8‐ to 12‐week‐old mice, 12‐ to 13‐month‐old mice presented more severe liver damage and higher liver inflammatory responses when exposed to 90 min of warm ischaemia and 8 h of reperfusion.^86^ Older mice showed decreased liver regeneration, as shown by decreased levels of HGF, c‐Met, cyclin D1, cyclin A2, proliferating cell nuclear antigen (PCNA) and SMP30, compared with young mice after PH. The dysfunction of liver regeneration and inhibition of autophagy in the older mice resulted in a decreased liver weight/body weight ratio and lower survival rate.^87^ Ageing livers undergoing hepatic I/R showed decreased mitophagy and depletion of Parkin in mice, while enhanced mitophagy more effectively protected livers against I/R injury.^88^ Liu et al. demonstrated that administration of plasma from young rats enhanced liver tissue restoration in old rats after hepatic I/R injury via activation of AMPK/ULK1‐mediated autophagy.^89^ The combination of ischaemia and rapamycin pretreatment protected old livers from I/R injury, as demonstrated by improved liver function and liver histology, and the protection was mediated by upregulation of autophagic flux in aged mice in vivo.^90^ It has been reported that one of the most important autophagy‐related proteins, ATG4β, is lost in aged livers, and overexpression of ATG4β reversed autophagy inhibition and subsequently reduced hepatic I/R injury in 26‐month‐old mice.^91^ This study shed light on autophagy enhancement in improving the prognosis of older patients undergoing PH or LT.

### 5.3 Autophagy regulation in steatotic liver grafts with hepatic I/R injury

Steatotic livers are more sensitive to liver resection or LT‐induced hepatic I/R, which results in an increased risk of postoperative complications.^92^, ^93^ Along with mitochondrial dysfunction, ER stress and inflammation after hepatic I/R, lipid accumulation and large cell volume, which obstructs the adjacent sinusoid space, result in reduced delivery of oxygen and nutrients, which contributes to hepatic I/R injury in steatotic livers.^94^ The impairment of autophagy in steatotic liver aggravated liver injury induced by cold I/R in steatotic rat livers, while pretreatment with the autophagic stimulator simvastatin effectively attenuated I/R‐induced liver injury. Simvastatin pretreatment effectively protects against microcirculation deterioration and endothelial dysfunction in moderately steatotic livers during cold storage and warm reperfusion via an NO‐mediated mechanism.^95^ On the other hand, the combined action of melatonin and trimetazidine significantly improved the liver function of steatotic liver grafts preserved in Institut Georges‐Lopez (IGL)‐1 solution via AMPK activation, autophagy activation and ER stress inhibition.^96^ With relevance to the prognosis of patients after LT and those with steatotic livers, hypothermic reconditioning (HR), which is implemented by insufflation of gaseous oxygen, notably improved graft function by attenuating mitochondrial dysfunction and restoring hepatocellular autophagy in rats.^97^ Domart et al^98^ demonstrated that IP before prolonged ischaemia decreased hepatocyte necrosis and liver dysfunction by activating autophagy and maintaining the ATP level in steatotic human livers. Moreover, IP inhibited hepatocellular necrosis and reduced the incidence of liver rejection via upregulation of autophagy in recipients who accepted steatotic grafts compared to recipients who accepted non‐IP steatotic grafts.^99^

## 6 CONCLUSION

In general, activation of autophagy is recognized as a mechanism that confers protection against hepatic I/R injury, while excessive autophagy is acknowledged for its role in accelerating the apoptosis of hepatocytes and/or liver nonparenchymal cells. It is widely accepted that stimulation of autophagy generally promotes liver regeneration and inhibits liver dysfunction, although the pathogenesis of warm and cold liver I/R injury is consistent with the complex interplay between autophagy, necrosis and apoptosis. Autophagy initiation is a potential mechanism to improve hepatocyte survival in patients after warm or cold I/R because it degrades intracellular components and eliminates organelle and protein waste. However, the results of studies on autophagy regulation during warm and/or cold liver I/R remain discordant. A fraction of studies documented downregulation of autophagy in protecting against warm or cold liver I/R injury via inhibition of inflammation and cellular apoptosis and necrosis. The dual role of autophagy suggests that upregulation or downregulation of autophagy may be an effective treatment strategy for the inhibition of liver damage. Autophagy regulation with gene modification in animal models provides a specific strategy to modify the expression of autophagic proteins and therefore is as an effective treatment to preserve liver function in hepatic I/R models. We suggest that future studies focus on clarifying the autophagy regulation mechanism in liver grafts from specific populations eligible for LT, which will contribute to increasing the LT rate and decreasing the low graft function rate in patients with end‐stage liver diseases.

## CONFLICT OF INTEREST

The authors declare no competing financial interests.

## AUTHOR CONTRIBUTIONS

**Chenxia Hu:** Validation (equal); Writing‐review & editing (lead). **Lingfei Zhao:** Writing‐review & editing (supporting). **Fen Zhang:** Validation (equal). **Lanjuan Li:** Conceptualization (lead).

## Data Availability

Not applicable.
